# Sitting-induced hemodynamic changes and association with sitting intolerance in children and adolescents: a cross-sectional study

**DOI:** 10.1038/s41598-020-70925-y

**Published:** 2020-08-18

**Authors:** Chunyan Tao, Zhenhui Han, Yongqiang Yan, Zhitao Pan, Hanwen Zhu, Xueying Li, Hongxia Li, Yuanyuan Wang, Ping Liu, Yuli Wang, Min Jiang, Chaoshu Tang, Hongfang Jin, Junbao Du

**Affiliations:** 1 Department of Pediatrics, Peking University First Hospital, No. 1, Xi’an-men Street, West District, Beijing, 100034 China; 2 Research Unit of Clinical Diagnosis and Treatment of Pediatric Syncope and Cardiovascular Diseases, Chinese Academy of Medical Sciences, No. 9, Dongdan Sanjo, Dongcheng District, Beijing, 100730 China; 3 Department of Cardiology, Children’s Hospital of Kaifeng, No. 87, Middle Section, Ziyou Road, Gulou District, Kaifeng, 475000 China; 4 Department of Pediatric Surgery, Children’s Hospital of Kaifeng, No. 87, Middle Section, Ziyou Road, Gulou District, Kaifeng, 475000 China; 5 Department of Medical Statistics, Peking University First Hospital, No. 1, Xi’an-men Street, West District, Beijing, 100034 China; 6 Department of Physiology and Pathophysiology, Peking University Health Science Centre, No. 38, Xueyuan Road, Haidian District, Beijing, 100191 China

**Keywords:** Cardiology, Diseases, Health care

## Abstract

Hemodynamic alteration with postural change from supine to sitting has been unclear in the young. In the cross-sectional study, 686 participants (371 boys and 315 girls, aged 6–18 years) were recruited from 4 schools in Kaifeng city, the central area of China. The active sitting test was performed to obtain heart rate (HR) and blood pressure (BP) changes from supine to sitting in children and adolescents. Hemodynamic change-associated sitting intolerance was analyzed. In the study participants, the 95th percentile (P_95_) values of changes in HR and BP within 3 min from supine to sitting were 25 beats/min and 18/19 mm Hg, respectively. Sixty-six participants had sitting intolerance symptoms. Compared with participants without sitting intolerance symptoms, those with symptoms more frequently had HR increase ≥ P_95_ or BP increase ≥ P_95_ within 3 min from supine to sitting (P < 0.001). Risk factors for sitting intolerance were age (odds ratio 1.218, 95% confidence interval 1.072–1.384, P = 0.002) and changes in HR or BP ≥ P_95_ within 3 min after sitting (odds ratio 2.902, 95% confidence interval 1.572–5.357, P = 0.001). We firstly showed hemodynamic changing profiles from supine to sitting and their association with sitting intolerance in children and adolescents. Sitting tachycardia is likely suggested with a change in HR ≥ 25 beats/min and sitting hypertension with a change in BP ≥ 20/20 mm Hg when changing from supine to sitting within 3 min. The age and changes in HR or BP were independent risk factors for sitting intolerance.

## Introduction

Lying, sitting and standing are inevitable postures in daily life, and blood distribution changes are accompanied by postural changes. Physiologically, changing from the supine to the orthostatic position induces an instantaneous and large shift of 10% to 25% of blood volume from the thorax to the lower extremities and splanchnic organs and a transfer from the vasculature to the interstitial tissues. This redistribution causes an immediate decrease in venous return to the heart, resulting in a transient decline in cardiac filling and blood pressure. Then, positive chronotropic and inotropic cardiac effects are stimulated by activation of the sympathetic nervous system and withdrawal of the parasympathetic nervous system to maintain a relatively stable circulatory condition, with an increase in heart rate of 10 to 20 beats/min, a negligible change in systolic blood pressure and a ~ 5 mm Hg increase in diastolic blood pressure^1,2^. Any abnormal regulation of the above process will lead to abnormal changes in heart rate and blood pressure, often characterizing individuals by orthostatic intolerance symptoms of syncope, dizziness, headache, palpitations, chest tightness, nausea, sweating, etc.^3–5^. In a consensus statement, Freeman et al. have suggested that the criterion of heart rate increment of postural orthostatic tachycardia syndrome is at least 40 beats/min in the young and elaborated the criterion of orthostatic hypotension^6^. Our epidemiologic study suggested the criterion of pediatric orthostatic hypertension^7^.

When changing from the supine position to sitting, we suppose that a similar hemodynamic alteration would occur, which likely results from gravity and regulatory mechanisms. The clinical clue is that sitting intolerance symptoms were observed in clinical practice, such as dizziness, palpitations, chest tightness, etc. elicited by sitting and relieved by recumbence^8,9^. Therefore, in children and adolescents, abnormal changes in heart rate and blood pressure might sometimes occur during sitting or the postural change from supine to sitting and be ambiguously associated with sitting intolerance which means that subjects are disturbed by the abovementioned symptoms during sitting or changing from supine to sitting. Unfortunately, up to now, no relevant studies have been conducted.

Abnormal orthostatic hemodynamic conditions lead to orthostatic intolerance symptoms and are risk factors for silent cerebrovascular diseases, ventricular hypertrophy, lacunar stroke or chronic kidney disease, etc., which always overstress patients and their families and cause future physiological harm^10–17^. As a result, the impact of abnormal sitting hemodynamic alteration on children and adolescents should not be neglected because of the similarity in pathogenesis and pathophysiology.

Therefore, this cross-sectional study was undertaken to focus on the changing hemodynamic characteristics from supine to sitting in children and adolescents aged 6 to 18 years and explore their possible associations with sitting intolerance.

## Methods

### Study design, setting and population

This cross-sectional study was conducted in Kaifeng city of Henan province, a central province in China. Four primary and middle schools where the students were arranged to get regular physical examinations were enrolled in this study. This study was approved by the Ethics Committee of Peking University First Hospital (No. 2018201) and the authority of each school. All methods in the study were carried out in accordance with the ethical standards expressed in the Declaration of Helsinki of 1964, as revised in 2000^18^. Informed consent was obtained from all subjects, or if subjects were under 18 years, from a parent or legal guardian who provided their medical history. Meanwhile, all subjects gave verbal assent and were also asked about their medical history and sitting intolerance symptoms such as dizziness, headache, blurred vision, palpitations and chest tightness with a change from the supine to the sitting position or prolonged sitting. Physical examinations including inspection, palpation, percussion and auscultation of heart, lung and abdomen were performed in the subjects. And their body weight and height were measured before the completion of active sitting test. Finally, 2 mL of venous blood was collected for blood routine test. All the procedures were conducted in quiet and comfortable rooms, but venipuncture was done in separate spaces where subjects were not able to go back and talk with the latter ones. Those who met the following conditions were excluded: (1) < 6 or > 18 years, (2) disturbed by chronic diseases such as myocarditis, hypertension, asthma, diabetes mellitus, renal dysfunction and epilepsy, (3) attacked by acute diseases such as acute upper respiratory tract infection (symptoms like fever, cough, sore throat, etc.) and acute gastroenteritis (symptoms like vomiting, nausea, abdominal pain, etc.), (4) taking drugs within 3 days prior to this study, (5) having no complete research data, or (6) unwilling to participate in this study or no informed consent gotten from their parents or guardians.

### Trained researchers

The researchers including pediatricians and postgraduates of pediatrics were trained in advance to understand the whole procedure of this study and know what should be asked and examined. Specific questionnaires were applied to collect medical history and sitting intolerance symptoms of subjects. For young children, especially for 6-year-old children, plain language was used to get information as accurately as possible. All the measurements were performed by the same research group.

### Anthropometric measures

Before measuring anthropometrics, the subjects were asked to remove their shoes, coats and hats. Body weight was measured with a digital scale and the reading was recorded when it became stable. Height was measured with a stadiometer. Body Mass Index was calculated as body weight (kg) divided by the square of height (m).

### Protocol for the active sitting test

After emptying their bladders, subjects underwent the active sitting test. The test was carried out in a quiet environment with a comfortable temperature of 22–24℃. Heart rate and blood pressure were monitored by using Dash 2000 Multi-Channel Physiologic Monitors (General Electric, Schenectady, New York, USA) which were equipped with different-sized cuffs according to the right upper arm circumferences of subjects. The facilities were calibrated with the mercury sphygmomanometer before the study. After a supine rest of at least 10 min and a stable heart rate (baseline heart rate) and blood pressure (baseline blood pressure) reached, heart rate was dynamically recorded and blood pressure was measured automatically. Then, subjects were asked to be seated upright in a chair with feet on the floor, knees bent at right angles, hands hanging down naturally and back leaning against nothing for another 10 min. Sitting heart rate and blood pressure were recorded every minute^19^. Any symptom complaint was noted. Subjects were not briefed on possible sitting intolerance symptoms and no leading questions were asked. Once the subject could not continue the test, it was terminated in advance.

### Calculation of sample size

The sample size was calculated with the data from our pilot study including 94 participants using NCSS_PASS 11.0 software (NCSS, Utah, USA) with confidence interval of 95% and with an alpha level of 0.05. A minimum sample size of 518 was supposed in the study.

### Statistical analyses

Statistical analyses were performed with SPSS 21.0 (IBM, New York, USA). Baseline characteristics are presented as mean ± standard error (SE) or n (%), and changes in heart rate and blood pressure are presented as percentiles, namely the 5th percentile (P_5_), the 10th percentile (P_10_), the 50th percentile (P_50_), the 90th percentile (P_90_) and the 95th percentile (P_95_). P_95_ was selected as a normal upper threshold^20^. Independent Student’s t test or Mann–Whitney U test was used to compare continuous variables between two groups according to the data distribution. Repeated-measures ANOVA was used to compare the values of heart rate and blood pressure over time during the active sitting test. The chi-square test was used to compare differences in categorical variables. Enter method of binary logistic regression analysis was used to analyze the independent association of variables with sitting intolerance. P < 0.05 indicated a statistical significance.

## Results

### Demographic characteristics

**Figure 1:**
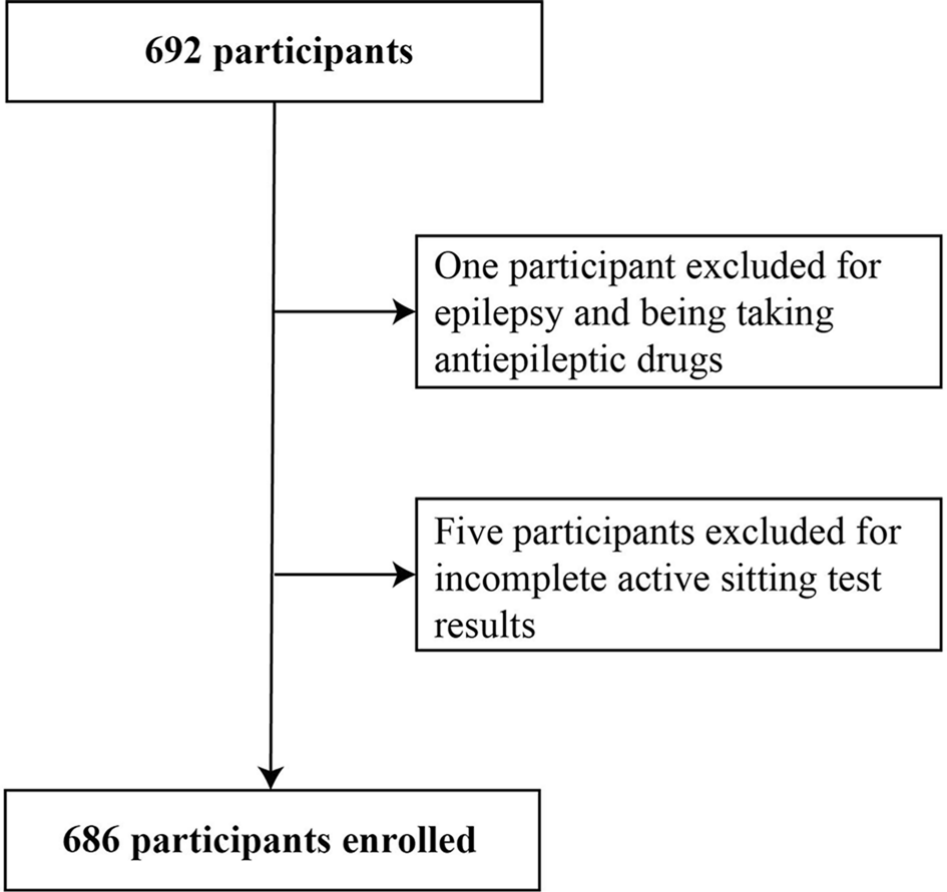
Flow chart of enrolling participants. Among the 692 participants, 1 was excluded for epilepsy and taking antiepileptic drugs, and another five were excluded for incomplete active sitting test results. 686 participants were finally recruited in the study.

In total, 686 participants were enrolled in this study after one being excluded for epilepsy and taking antiepileptic drugs, as well as five being excluded for incomplete active sitting test results (low compliance) (Fig. 1); 371 were boys (54.1%) and 315 were girls (45.9%). The mean age was 11.4 ± 0.1 years.

### Hemodynamic changes during the active sitting test

**Figure 2:**
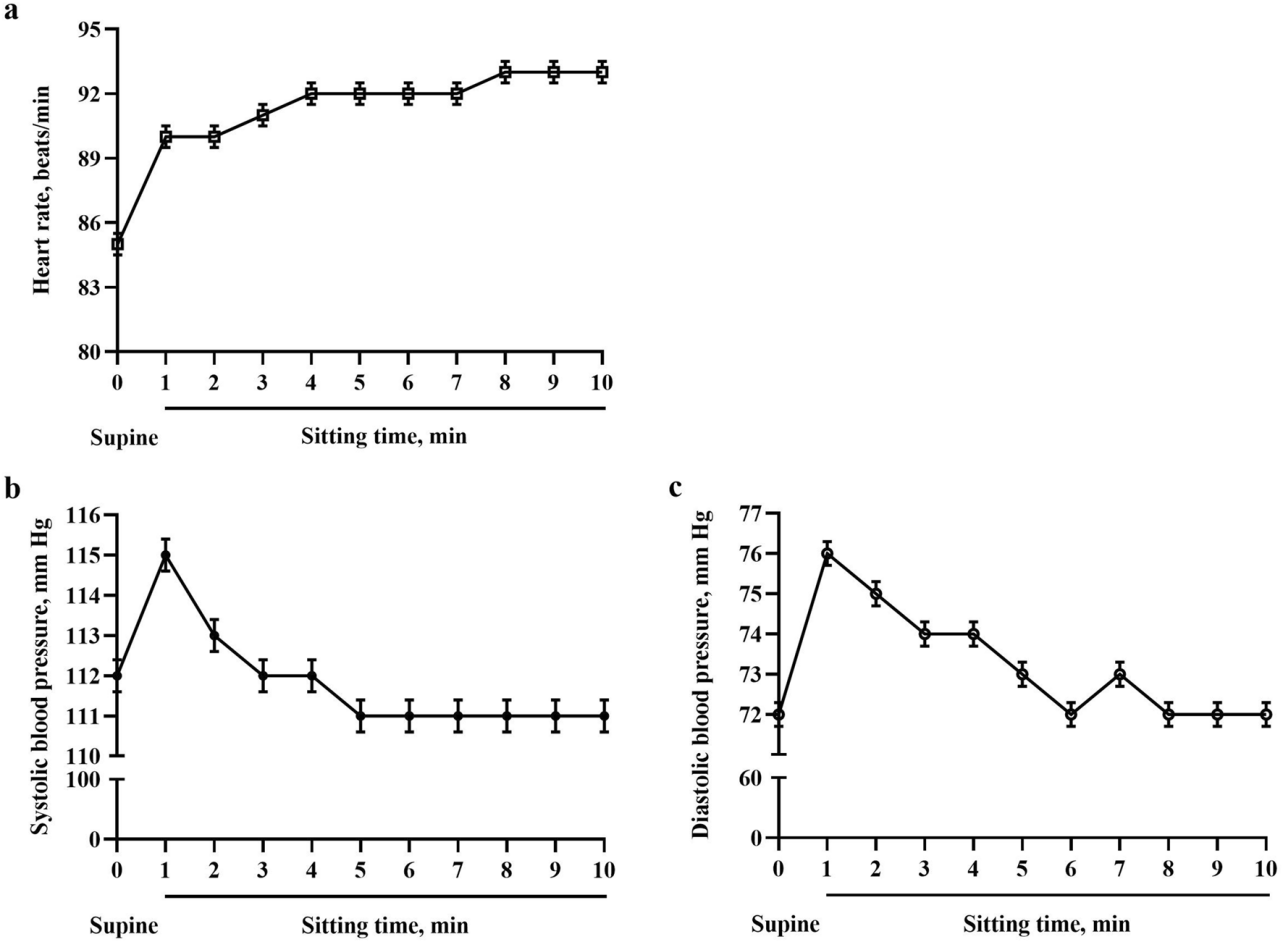
Changes in hemodynamics for children and adolescents in active sitting test. Changes in (**a**) heart rate, (**b**) systolic blood pressure and (**c**) diastolic blood pressure. Data are mean ± SE. Heart rate and blood pressure changed promptly with postural change from supine to sitting and then reached a relatively stable condition.

For all participants, the baseline heart rate was 85 ± 0.5 beats/min, and the heart rate after sitting increased quickly to a relatively stable level, showing a significant difference between baseline heart rate and that while sitting (P < 0.001, Fig. 2a). The systolic blood pressure within 2 min after sitting and diastolic blood pressure within 4 min after sitting were higher than those in the supine position (P < 0.001). Then, the other values at other time intervals after sitting did not differ from supine values (P > 0.05, Fig. 2b,c).

The percentiles (P_5_, P_10_, P_50_, P_90_ and P_95_) of changes in heart rate and systolic and diastolic blood pressure during the active sitting test (maximum heart rate and systolic and diastolic blood pressure within any duration after sitting minus the baseline values) are listed in Table 1. It showed that most participants had a gradual increase in heart rate during sitting, but were characterized with a relatively stable change in blood pressure.

**Table 1:** Percentiles of changes in heart rate and blood pressure between the supine position and sitting position.

Items	P_5_	P_10_	P_50_	P_90_	P_95_
∆HR1, beats/min	− 10	− 6	4	18	22
∆HR2, beats/min	− 6	− 2	7	20	24
∆HR3, beats/min	− 3	0	9	21	25
∆HR4, beats/min	− 2	1	10	22	26
∆HR5, beats/min	− 2	2	11	23	27
∆HR6, beats/min	− 1	2	12	23	29
∆HR7, beats/min	− 1	3	12	24	29
∆HR8, beats/min	0	3	13	25	30
∆HR9, beats/min	0	4	14	25	30
∆HR10, beats/min	0	4	14	25	30
∆SBP1/∆DBP1, mm Hg	− 9/− 8	− 6/− 5	3/4	13/13	15/17
∆SBP2/∆DBP2, mm Hg	− 7/− 5	− 3/− 3	5/6	14/14	17/17
∆SBP3/∆DBP3, mm Hg	− 6/− 4	− 3/− 2	5/7	14/15	18/19
∆SBP4/∆DBP4, mm Hg	− 5/− 3	− 2/− 1	6/7	14/15	18/19
∆SBP5/∆DBP5, mm Hg	− 5/− 3	− 2/0	6/7	14/16	18/20
∆SBP6/∆DBP6, mm Hg	− 4/− 3	− 2/0	6/7	15/16	19/20
∆SBP7/∆DBP7, mm Hg	− 4/− 2	− 2/0	6/8	15/16	19/20
∆SBP8/∆DBP8, mm Hg	− 4/− 2	− 2/0	7/8	15/17	19/20
∆SBP9/∆DBP9, mm Hg	− 4/− 2	− 2/1	7/8	16/18	19/20
∆SBP10/∆DBP10, mm Hg	− 4/− 2	− 1/1	7/8	16/17	19/21

∆HR means the maximum heart rate within a relevant duration after sitting minus the supine heart rate. ∆SBP/∆DBP means the maximum systolic blood pressure and diastolic blood pressure within a relevant duration after sitting minus the corresponding values in the supine position.

*HR* heart rate, *SBP* systolic blood pressure, *DBP* diastolic blood pressure.

### Sitting intolerance and its association with hemodynamic changes

Among 686 participants, 66 (9.6%) had sitting intolerance symptoms, dizziness being the most frequent (49/66, 74%), followed by blurred vision (14/66, 21%), headache (10/66, 15%), chest tightness (6/66, 9%), nausea (4/66, 6%), abdominal pain (2/66, 3%), numbness (2/66, 3%) and sweating (1/66, 2%). Participants with sitting intolerance had a similar sex ratio and similar baseline heart rate and blood pressure to those without sitting intolerance (P > 0.05), but those with sitting intolerance had increased age, weight, height and body mass index (P < 0.05, Table 2). With P_95_ of changes in heart rate and blood pressure as the upper threshold, the incidence of sitting intolerance in participants with heart rate change ≥ P_95_ within 2 or 3 min after sitting was significantly higher than those with heart rate change < P_95_ (P < 0.05). Sitting intolerance incidence did not differ between participants with heart rate change ≥ P_95_ and < P_95_ during any other durations after sitting (P > 0.05). Simultaneously, sitting intolerance incidence was higher for those with systolic and/or diastolic blood pressure change ≥ P_95_ than < P_95_ within any time after sitting (P < 0.05). In accordance with the suggested time after standing in diagnosing orthostatic hypertension or orthostatic hypotension, 3 min after sitting was selected as an optimal duration applied in clinical practice^7,21^. Heart rate increase ≥ P_95_ or blood pressure increase ≥ P_95_ within 3 min from supine to sitting was more frequent for participants with than without sitting intolerance symptoms (P < 0.001, Table 2).

**Table 2:** Comparison of baseline characteristics for participants with and without sitting intolerance.

Items	With sitting intolerance	Without sitting intolerance	χ^2^/Z/t value	P value
Cases, n (%)	66 (9.6%)	620 (90.4%)	−	−
Sex, n (M/F)	36/30	336/285	0.006	0.937
Age, years	12.3 ± 0.2	11.4 ± 0.1*	− 3.516	< 0.001
Weight, kg	44.0 ± 1.5*	39.0 ± 0.5*	− 3.327	0.001
Height, cm	150.8 ± 1.5	145.5 ± 0.5*	− 3.144	0.002
Body mass index, kg/m^2^	19.1 ± 0.4*	18.1 ± 0.1*	− 2.733	0.006
Supine heart rate, beats/min	84 ± 1.5	85 ± 0.5*	− 1.099	0.272
Supine systolic blood pressure, mm Hg	113 ± 1.1	112 ± 0.4	0.881	0.379
Supine diastolic blood pressure, mm Hg	72 ± 1.1	72 ± 0.3	0.602	0.547
Heart rate increase ≥ P_95_ or blood pressure increase ≥ P_95_ within 3 min after sitting, n (yes/no)	18/48	68/552	14.463	< 0.001

Data are mean ± SE.

*M* male, *F* female.

*Continuous variables with non-normal distribution.

### Independent risk factors for sitting intolerance

Variables with P < 0.05 in the univariate analysis except weight and height which were used to calculate body mass index, were included in the binary logistic regression analysis, and the results showed that age and changes in heart rate or blood pressure were independent risk factors for sitting intolerance (P < 0.05, Table 3). For each 1-year increase in age, the probability of sitting intolerance was increased by 21.8%, and the participants with the changes in heart rate or blood pressure ≥ P_95_ within 3 min after sitting had the likelihood of sitting intolerance about 2 times higher than those without such changes.

**Table 3:** Binary logistic regression analysis for possible risk factors for sitting intolerance.

Variables	B	SE	Wald	P value	Odds ratio (95% confidence interval)
Age, years	0.197	0.065	9.196	0.002	1.218 (1.072–1.384)
Changes in heart rate or blood pressure ≥ P_95_ within 3 min after sitting, yes/no	1.066	0.313	11.611	0.001	2.902 (1.572–5.357)
Body Mass Index, kg/m^2^	0.046	0.040	1.314	0.252	1.047 (0.968–1.133)

### Stratified analysis of hemodynamic changes and sitting intolerance

To control the confounding effect of age, the participants were stratified as ≤ 12 years (n = 437) and > 12 years (n = 249). We found that in participants ≤ 12 years, there was a significant association between changes in heart rate or blood pressure ≥ P_95_ within 3 min after sitting and sitting intolerance (odds ratio = 4.450, 95% confidence interval 2.061–9.611, P < 0.001). However, in participants > 12 years, changes in heart rate or blood pressure ≥ P_95_ within 3 min after sitting were not associated with sitting intolerance (odds ratio = 1.856, 95% confidence interval 0.694–4.964, P = 0.218).

## Discussion

For the first time, we showed the hemodynamic changing profiles from supine to sitting in children and adolescents and their association with sitting intolerance. In young people, sitting tachycardia is likely suggested with a change in heart rate ≥ 25 beats/min and sitting hypertension with a change in blood pressure ≥ 20/20 mm Hg (for easy memory) when changing from supine to sitting within 3 min. The age and changes in heart rate or blood pressure were independent risk factors for sitting intolerance. Furthermore, age stratification analysis showed that only in aged ≤ 12-year-old population, changes in heart rate or blood pressure ≥ P_95_ within 3 min after sitting were risk factors for sitting intolerance.

Exact hemodynamic changes from supine to sitting were not clearly known previously, but they were presumed to be approximately consistent with changes caused by gravity from the supine to standing position, and both likely have similar regulatory mechanisms^22,23^. In our study, we found an average increase in heart rate in active sitting test of 4–14 beats/min and an average increase in blood pressure of 3–7/4–8 mm Hg, showing a similar trend to changes from the supine to upright position.

Reduced stimulus to baroreceptors, leading to decreased venous return to the heart, activates the sympathetic nervous system and withdraws parasympathetic nervous activity for a compensatory increase of heart rate, myocardial contractility and vascular tone; thus, normal blood pressure and organic perfusion can be maintained^24–26^. The above progressive regulation will be accomplished in a short period of time. As time goes on, humoral regulation is also thought to participate in this process, especially with long postural alterations^27^. In our children and adolescents, heart rate and blood pressure changed promptly after postural alteration and then reached a relatively stable condition. Failure of any regulatory mechanisms will result in abnormal changes in heart rate and blood pressure, probably accompanied by sitting intolerance symptoms. Sympathetic hyperactivity, hypovolemia, endothelial dysfunction, muscle pump defects, etc. are the possible mechanisms of postural orthostatic tachycardia syndrome and orthostatic hypertension/hypotension^19,28–31^. From our analysis, these factors might also play important roles in regulating abnormal sitting hemodynamics.

In the present study, sitting tachycardia and sitting hypertension were both related to sitting intolerance. We found no correlation between sitting hypotension (changes in blood pressure ≤ P_5_, taking P_5_ as a normal lower threshold) and sitting intolerance. The prevalence of orthostatic hypotension in young people is considered low because it increases with age, and the prevalence in adults is about 5%^32^. Theoretically, a lesser amount of blood is transferred from the thorax to the lower extremities and splanchnic organs during sitting than in the upright position, and as a result, the decrease in sitting blood pressure is not obvious as compared with the decrease in upright blood pressure. The relation between sitting hypotension and sitting intolerance merits further exploration in large populations.

Additionally, we found that the increase in age is an independent risk factor for sitting intolerance; indeed, older children are more often troubled by postural change from supine to upright or prolonged standing than younger children^33^. The imbalance of neurohumoral regulation might be the explanation^34^.

As the negative impact of abnormal orthostatic hemodynamics on human body, abnormality of sitting hemodynamics is presumed to be harmful. Researchers found that prolonged sitting had adverse impact on vascular function in young females and they were generally aware that adjustment between sedentary behaviors (e.g. prolonged sitting) and physical exercise could affect the control of blood pressure^35–37^.

This study still has limitations. Our study population was from the central area of China, but whether the results are applicable to the children and adolescents in other areas needs further validation. Moreover, the actual impact of hemodynamic changes from supine to sitting on the human body is not fully understood, and large longitudinal studies should be conducted.

## Conclusion

In summary, hemodynamic changes occur with a position shift from supine to sitting in children and adolescents; those with remarkable changes are accompanied by sitting intolerance symptoms. Based on the present findings, we suggested that in young people, sitting tachycardia is likely suggested with a change in heart rate ≥ 25 beats/min and sitting hypertension with a change in blood pressure ≥ 20/20 mm Hg when changing from supine to sitting within 3 min. The present study furthers our understanding of sitting-induced hemodynamic changes and provides useful data for establishing possible criteria of pediatric sitting tachycardia and sitting hypertension. Large sample-sized studies are needed to investigate risk factors and possible preventive measures for abnormal sitting-induced hemodynamic changes in young people and their impact on targeted organs.

## Data Availability

The dataset generated during the current study is available from the corresponding authors upon reasonable request.

## References

[1] Smith JJ, Porth CM, Erickson M (1994). Hemodynamic response to the upright posture. J. Clin. Pharmacol..

[2] Persson PB (1996). Modulation of cardiovascular control mechanisms and their interaction. Physiol. Rev..

[3] Garland EM, Celedonio JE, Raj SR (2015). Postural tachycardia syndrome: Beyond orthostatic intolerance. Curr. Neurol. Neurosci. Rep.

[4] Raj SR (2013). Postural tachycardia syndrome (POTS). Circulation.

[5] Kario K (2009). Orthostatic hypertension: A measure of blood pressure variation for predicting cardiovascular risk. Circ. J..

[6] Freeman R (2011). Consensus statement on the definition of orthostatic hypotension, neurally mediated syncope and the postural tachycardia syndrome. Clin. Auto Res..

[7] Zhao J (2015). A cross-sectional study on upright heart rate and BP changing characteristics: Basic data for establishing diagnosis of postural orthostatic tachycardia syndrome and orthostatic hypertension. BMJ Open.

[8] Khadilkar SV, Yadav RS, Jagiasi KA (2013). Are syncopes in sitting and supine positions different? Body positions and syncope: A study of 111 patients. Neurol. India.

[9] Ikeda T, Ohbuchi H, Ikenoue T, Mori N (1992). Maternal cerebral hemodynamics in the supine hypotensive syndrome. Obstet. Gynecol..

[10] Benrud-Larson LM, Dewar MS, Sandroni P, Rummans TA, Haythornthwaite JA, Low PA (2002). Quality of life in patients with postural tachycardia syndrome. Mayo Clin. Proc..

[11] Raj V (2009). Psychiatric profile and attention deficits in postural tachycardia syndrome. J. Neurol. Neurosurg. Psychiatry.

[12] Thomas RJ, Liu K, Jacobs DRJR, Bild DE, Kiefe CI, Hulley SB (2003). Positional change in blood pressure and 8-year risk of hypertension: The CARDIA study. Mayo Clin. Proc..

[13] Kario K (2007). Preceding linkage between a morning surge in blood pressure and small artery remodeling: An indicator of prehypertension. J. Hypertens..

[14] Fan XH (2010). Disorders of orthostatic blood pressure response are associated with cardiovascular disease and target organ damage in hypertensive patients. Am. J. Hypertens..

[15] Hoshide S (2008). Orthostatic hypertension detected by self-measured home blood pressure monitoring: A new cardiovascular risk factor for elderly hypertensives. Hypertens. Res..

[16] Masoud M, Sarig G, Brenner B, Jacob G (2008). Orthostatic hypercoagulability: A novel physiological mechanism to activate the coagulation system. Hypertension.

[17] Gorelik O, Cohen N (2015). Seated postural hypotension. J. Am. Soc. Hypertens..

[18] A fifth amendment for the Declaration of Helsinki. *Lancet* **356**, 1123 (2000). 11030284

[19] Hoshide S, Parati G, Matsui Y, Shibazaki S, Eguchi K, Kario K (2012). Orthostatic hypertension: Home blood pressure monitoring for detection and assessment of treatment with doxazosin. Hypertens. Res..

[20] Dong Y (2017). National blood pressure reference for chinese han children and adolescents aged 7 to 17 years. Hypertension.

[21] Brignole M (2018). 2018 ESC guidelines for the diagnosis and management of syncope. Eur. Heart J..

[22] Ogoh S, Fadel PJ, Monteiro F, Wasmund WL, Raven PB (2002). Haemodynamic changes during neck pressure and suction in seated and supine positions. J. Physiol..

[23] Shaw BH, Loughin TM, Machey DC, Robinovitch SN, Claydon VE (2014). The effect of orthostatic stress type on cardiovascular control. Blood Press. Monit..

[24] Sanders JS, Mark AL, Ferguson DW (1989). Importance of aortic baroreflex in regulation of sympathetic responses during hypotension. Evidence from direct sympathetic nerve recordings in humans. Circulation.

[25] Izzo JLJR, Taylor AA (1999). The sympathetic nervous system and baroreflexes in hypertension and hypotension. Curr. Hypertens. Rep..

[26] Ichinose M, Nishiyasu T (2012). Arterial baroreflex control of muscle sympathetic nerve activity under orthostatic stress in humans. Front. Physiol..

[27] Nilsson D, Sutton R, Tas W, Burri P, Melander O, Fedorowski A (2015). Orthostatic changes in hemodynamics and cardiovascular biomarkers in dysautonomic patients. PLoS ONE.

[28] Jarjour IT (2013). Postural tachycardia syndrome in children and adolescents. Semin. Pediatr. Neurol..

[29] Jones PK, Shaw BH, Raj SR (2016). Clinical challenges in the diagnosis and management of postural tachycardia syndrome. Pract. Neurol..

[30] Kario K (2002). U-curve relationship between orthostatic blood pressure change and silent cerebrovascular disease in elderly hypertensives: Orthostatic hypertension as a new cardiovascular risk factor. J. Am. Coll. Cardiol..

[31] Kario K, Mitsuhashi T, Shimada K (2002). Neurohumoral characteristics of older hypertensive patients with abnormal nocturnal blood pressure dipping. Am. J. Hypertens..

[32] Fagard RH, De CP (2010). Orthostatic hypotension is a more robust predictor of cardiovascular events than nighttime reverse dipping in elderly. Hypertension.

[33] Kenny RA, Bhangu J, King-Kallimanis BL (2013). Epidemiology of syncope/collapse in younger and older Western patient populations. Prog. Cardiovasc. Dis..

[34] Wang C (2018). 2018 Chinese Pediatric Cardiology Society (CPCS) guideline for diagnosis and treatment of syncope in children and adolescents. Sci. Bull..

[35] McManus AM (2015). Impact of prolonged sitting on vascular function in young girls. Exp. Physiol..

[36] Dempsey PC, Larsen RN, Dunstan DW, Owen N, Kingwell BA (2018). Sitting less and moving more: Implications for hypertension. Hypertension.

[37] Armstrong, V. The impact of shear rate and prolonged sitting on endothelial function in children. (Doctoral dissertation, University of British Columbia, 2015).

